# Spatiotemporal analysis of canine rabies in El Salvador: Violence and poverty as social factors of canine rabies

**DOI:** 10.1371/journal.pone.0201305

**Published:** 2018-08-17

**Authors:** Patricia Arias-Orozco, Fernando Bástida-González, Lilian Cruz, Jacqueline Villatoro, Eduardo Espinoza, Paola Berenice Zárate-Segura, Sergio Recuenco

**Affiliations:** 1 Laboratorio de Medicina Traslacional, Escuela Superior de Medicina, Instituto Politécnico Nacional, St. Salvador Díaz Mirón Esquina Plan de San Luis, Santo Tomas, Miguel Hidalgo, México; 2 Laboratorio de Biología Molecular, Laboratorio Estatal de Salud Pública del Estado de México, Paseo Tollocan s/n, Col. La Moderna de la Cruz, Toluca, Mexico; 3 Unidad de Zoonosis, Laboratorio Nacional de Referencia, Ministerio de Salud, San Salvador, El Salvador; 4 Ministerio de Salud de El Salvador, San Salvador, El Salvador; 5 Centro Nacional de Salud Pública, INS, Lima, Perú; Wistar Institute, UNITED STATES

## Abstract

**Background:**

The incidence of canine rabies cases in El Salvador has decreased in the last decade since the establishment of intense control programs, such as massive vaccination campaigns implemented by the Ministry of Health. Socioeconomic crises in recent years have limited the access to certain areas across the country and have impacted surveillance and prevention campaigns, which places the country at risk for a resurgence of canine rabies.We aimedto describe the spatiotemporal patterns of canine rabies and its association with critical social factors in El Salvador from 2005 to 2014.

**Method:**

We included 459 cases of canine rabies. Several socioeconomic, demographic, and surveillance variables were modeled using a Poisson regression to evaluate their associations with the incidence of canine rabies. Spatial scan statistics were adjusted or unadjusted with covariates and applied to identify statistically significant clusters of canine rabies. Finally, a canine rabies risk map was created.

**Results:**

A positive association and higher risk of canine rabies were found for low poverty zones, where it is suspected that urban slums contribute to ongoing rabies transmission (RR = 7.74). Violence had a negative association with rabies (RR = 0.663), which is likely due to reporting bias. Significant clusters were identified in all five epidemiological regions, and the Eastern region had the highest risk (RR = 50.62). The influences of the selected variables in cluster detection were confirmed by the adjusted analysis. Higher-risk townships were distributed from the Western to the Eastern regions of the country.

**Conclusion:**

Social factors are determinants of rabies in El Salvador and play a major role in national spatial patterns of the disease. There are high-risk areas for canine rabies across the country, and there were two persistent rabies foci during the study period. Examining the role of social factors can provide better insight into rabies in vulnerable countries, and socioeconomic factors can be key elements in developing better policies and interventions for rabies control.

## Introduction

Rabies is a globally distributed zoonotic viral disease that affects the central nervous system, causing encephalitis and death. The World Health Organization (WHO) estimates there are 61,000 human deaths worldwide per year [1, 2]. Dogs are the primary source of human infection. Controlling canine rabies is essential for effectively preventing transmission to humans [3, 4]. The incidence of canine rabies has declined in most Latin American countries since government health agencies began implementing rabies control programs in the 1990s [5, 6]. Furthermore, in El Salvador, the Ministry of Health observed a reduction in canine rabies cases between 2005 and 2015, and no new human rabies cases have been reported since 2008 [7].

The economic and social crises faced by El Salvador in recent decades [8] may be hindering new and ongoing canine rabies control activities in some areas, which could possibly lead to a resurgence in rabies cases and underestimation of the true disease burden [1, 2]. El Salvador has one of the highest murder rates in Latin America and in the world [8, 9]. This violence is associated with the expansion of criminal gangs in the country since the end of the civil war in 1992. These gangs, or “*Maras*,” originated in the United States with Salvadoran immigrants during the 1980s. Gang members who were deported from the USA returned to El Salvador, initially establishing themselves in marginalized, poor, urban areas of San Salvador, the capital city, and spreading throughout the country [10]. Gangs usually take control of territories and restrict free transit for authorities and the regular population, making control activities risky and more difficult to implement [11]. The issues of violence are not isolated and come with major issues such as economic inequality, poverty, overcrowding, illiteracy, and social exclusion [12, 13].

The prevalence of canine rabies has been associated with developing countries and poorer rural and urban areas where health quality and services are limited [1, 5]. Furthermore, the Commission on Social Determinants of Health (WHO 2008) stated that social factors have an influence on health inequities. It is important to identify social determinants to improve and better address vulnerable populations [14, 15]. In this context, the aim of this study is to describe the spatial distribution of canine rabies in El Salvador during 2005–2014 and to look for an association with three critical social determinants in the country: poverty, violence, and illiteracy. The findings could be useful for decision makers responsible for the development of control and prevention strategies for canine rabies in areas with social conflict.

## Methods

### Study area

**Fig 1:**
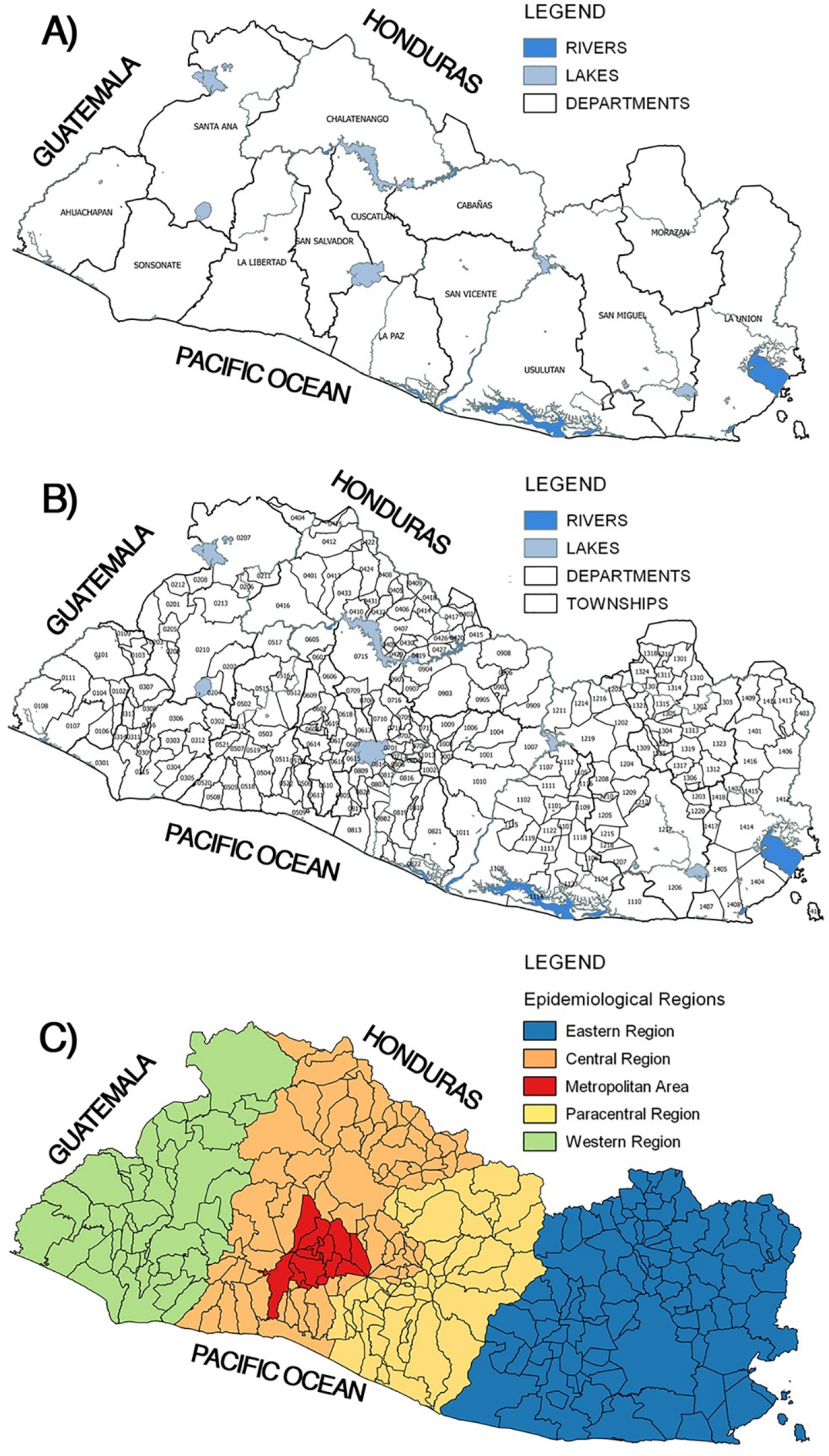
Official territorial division of El Salvador. A) Administrative department division; B) Townships; C) Epidemiological region: metropolitan area (red; San Salvador), Western region (green; Santa Ana, Ahuachapan, and Sonsonate), Central region (orange; Chalatenango, Cuscatlan, and La Libertad), Paracentral region (yellow; Cabañas, San Vicente and La Paz) and Eastern region (Blue; Usulutan, San Miguel, Morazan, and La Union) [17]. Use of data authorized by Geoportal-CNR under a CC BY license, with permission from Geoportal-CNR, original copyright 2014.

The study examined the country of El Salvador, which is divided into 14 departments with 262 townships and has total surface area of 21,040.90 km^2^. For the analyses, the country was divided into five epidemiological regions: Eastern, Paracentral, Metropolitan, Central, and Western (Fig 1). A national population of 5,734,000 people was estimated from the 2007 census [16].

### Independent variables

Seven independent variables were defined, including three social variables (violence, poverty, and illiteracy), two demographic variables (canine and human population density), and two surveillance variables (the number of public health care centers and canine rabies vaccination coverage). The murder rate was used as proxy of violence, with four ranges grouped in quartiles and coded as a dummy variable. The first range includes values of <10 murders per 100,000 people and was set according to the WHO’s threshold for an epidemic of 10 murders per 100,000 people.

The poverty variable was included because of its relationship with the incidence of canine rabies in developing countries [1, 5]. The illiteracy rate was used as an indicator for the level of education [18]. Regarding demographic factors, canine and human population densities were considered due to their relevance in the transmission of rabies among animal and human populations [19, 20]. The number of public health centers per geographic unit was used to increase the sensitivity of the analysis [21, 22]. Canine rabies vaccinations were included in the study as a mandatory variable because it is a standard indicator of success when evaluating canine rabies control [21].

### Data collection and management

Rabies surveillance data were obtained from the El Salvador Ministry of Health. Laboratory-confirmed cases of canine rabies reported during the period of 2005–2014 were aggregated at the township level for the analysis. The diagnoses were performed by health workers in the Unit of Zoonotic Disease using mouse inoculation and a direct fluorescent antibody test (DFA).

For spatial analysis, the centroid geographical coordinates for each township were obtained with QGIS software in the WSG84_16N metric geographic system. Spatial data were obtained from the National Records Center, Geoportal, El Salvador [17]. Polygon and point maps were created with ArcGIS 10.2 software. All laboratory-confirmed positive cases of canine rabies were georeferenced, and a kernel density analysis was performed for a smooth display of the areas with the highest concentration of canine rabies during the study period [23]. Vaccination coverage was obtained from the El Salvador Ministry of Health, who calculate the percentage of dog vaccinations with respect to the expected number of dogs and cats and the number of vaccinated dogs per department (S5 Fig).

The variables included in the analysis were available from different data sources. Illiteracy and demographic data were obtained from the DIGESTYC and the Institute of Statistics Reports [16]. Township poverty classifications were acquired from the Local Development Investment Fund (FISDL) [24]. This document classifies poverty by township as low, mid-low, mid-high, and high poverty based on two indicators: economic income and unmet basic needs. Murder rates were obtained from the FUNDAUNGO foundation and official statistics of the judiciary branch of El Salvador [25, 26]. Finally, the canine population was estimated using canine/human indicators provided by national health authorities. This information is sorted by epidemiological region and township.

### Statistical analysis

A Poisson regression model was used to evaluate the association of canine rabies with demographic and social variables [27, 28]. A database was constructed, which included 262 townships over a 10-year period. The analysis unit was defined as township-years, which was calculated as the number of townships (262) multiplied by the number of years in the study (10) for a total of 2,620 township-years. The dependent variable was the observed number of positive cases of canine rabies in each township for a given year, with the townships’ area as an offset variable. The response variable was defined as the expected canine rabies in each township for a given year.

Seven independent variables were defined: violence, poverty, illiteracy, canine population density, human population density, number of public health centers, and vaccination coverage. The model analysis was performed in STATA 13.1 software [29]. An adjustment for overdispersion was performed with the SCALE (x2) command [28]. Model fit was assessed with the deviance and p value.

### Cluster analysis

A spatial scan statistic was used to detect statistically significant clusters of canine rabies for each year using the purely spatial Poisson model developed by Kuldorff [30, 31]. Canine rabies cases were aggregated at the centroid of each geographical study unit. A cluster was considered statistically significant when the p value was ≤ 0.05, which was modeled using 999 Monte Carlo replications [31]. The maximum size for the mobile window of the scan was set as 20% of the population at risk with an elliptical shape [32]. All cluster analyses were performed in SaTScan™ v8.0 software [30]. The effect of covariates on the spatial clustering of canine rabies was evaluated with an adjusted analysis using the Poisson model. This was done to control for the effect of variables and identify persistent clusters that are not due to the combination of variables defined in the study [31, 33].

### Risk assessment

The risk of canine rabies cases was expressed as the expected cases of canine rabies in each township per km^2^. Expected cases of canine rabies were estimated with a Poisson regression adjusted for overdispersion including the independent variables mentioned above. The risk was stratified by quartiles. The model equation was:

$$\log(EXP)=\ln\left(Area\right)+(\beta_{0}+\beta_{1}Canine\;{Density}_{1}+\beta_{2}Human\;{Density}_{2}+\beta_{3}Public\;Health\;care\;centers+\beta_{4}Vacctination\;Coverage+\beta_{5}Illiteracy+\beta_{6}Poverty+\beta_{2}Insecurity)+\varepsilon_{i}$$ (1)

## Results

### Canine rabies in El Salvador

**Fig 2:**
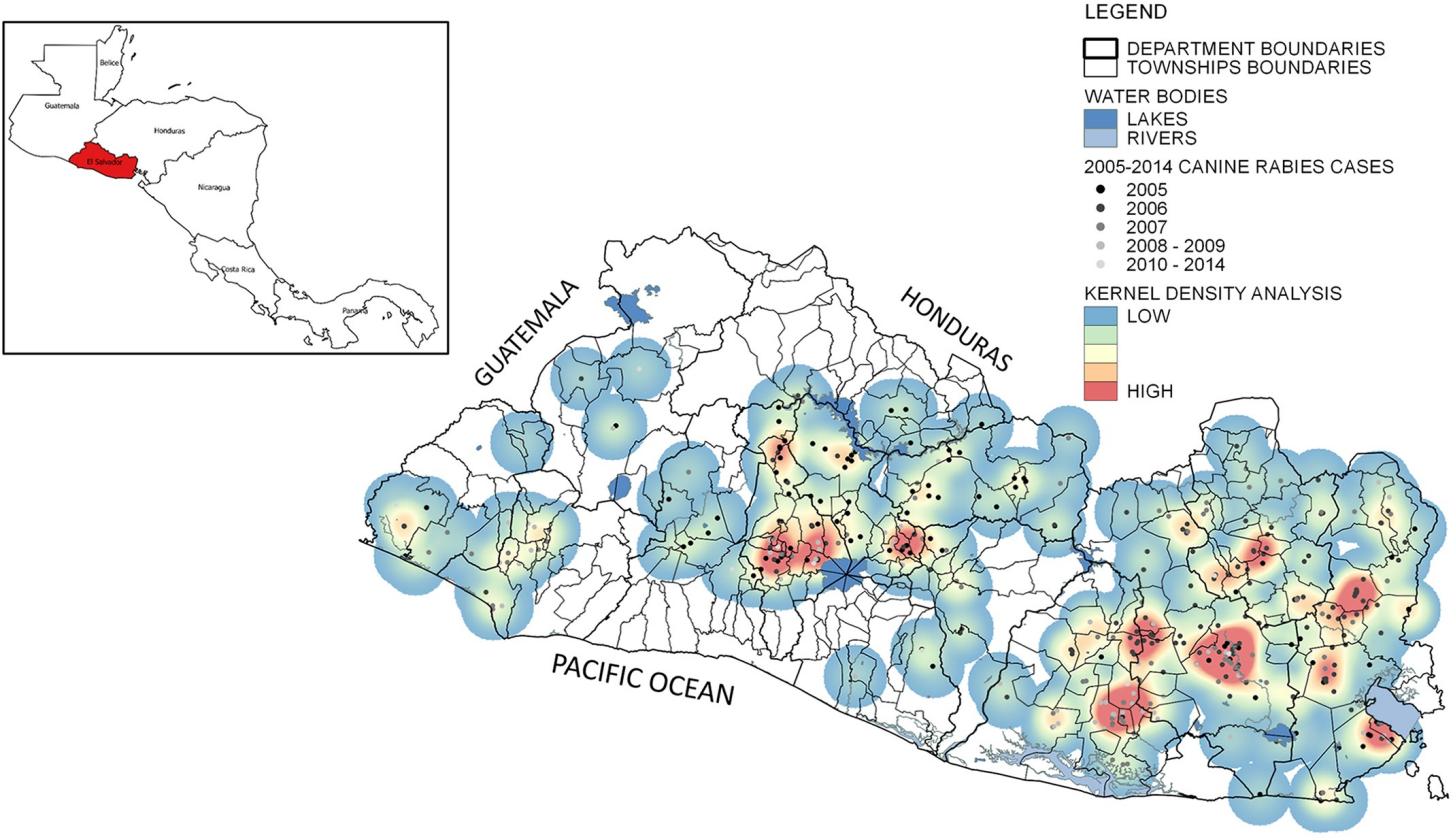
Laboratory-confirmed positive canine rabies cases, 2005–2014, and Kernel Density analysis in El Salvador. The highest number of positive canine rabies cases was reported in the Eastern region. Hot spots were identified in all five epidemiological regions. Source: Ministry of Health, El Salvador. Use of data authorized by Geoportal-CNR under a CC BY license, with permission from Geoportal-CNR, original copyright 2014.

A total of 2,131 dogs were tested at the National Reference Laboratory in El Salvador during the study period. The Metropolitan area (San Salvador City) submitted the largest percentage of specimens of 28.6% (623), and the Western region reported the lowest percentage of 8.8% (193). A total of 459 dogs tested positive for rabies. The largest percentage of positive cases of canine rabies was reported in 2006, with 84.2% (177 cases) located in the Eastern Region, and 99 of those cases were reported in the San Miguel Department (Table 1). Areas with higher density of positive canine rabies cases were identified in the Central, Paracentral, Eastern, and Metropolitan regions in the kernel density analysis (Fig 2).

**Table 1:** Annual number and percentage of positive canine rabies cases from five epidemiological regions in El Salvador in 2005–2014.

			REGION			
YEAR	Western	Central	Metropolitan area	Paracentral	Eastern	TOTAL
**2005**						
Samples^a^	28	75	123	56	55	337
Cases^b^	8	31	23	19	47	128
%^c^	6.25	24.22	17.97	14.84	36.72	100
**2006**						
Samples^a^	10	31	83	32	206	362
Cases^b^	3	5	16	4	149	177
%^c^	1.69	2.82	9.04	2.26	84.18	100
**2007**						
Samples^a^	32	27	96	46	97	298
Cases^b^	11	4	4	10	55	84
%^c^	13.10	4.76	4.76	11.90	65.48	100
**2008**						
Samples^a^	28	32	74	24	98	256
Cases^b^	10	4	5	2	21	42
%^c^	23.81	9.52	11.90	4.76	50.00	100
**2009**						
Samples^a^	18	18	50	21	95	202
Cases^b^	1	1	1	0	15	18
%^c^	5.56	5.56	5.56	0	83.33	100
**2010**						
Samples^a^	23	17	54	18	51	163
Cases	2	0	2	0	1	5
%^c^	40	0	40	0	20	100
**2011**						
Samples^a^	21	16	47	13	74	171
Cases^b^	0	1	0	0	1	2
%	0	50	0	0	50	100
**2012**						
Samples^a^	9	11	34	14	75	143
Cases^b^	0	0	1	1	0	2
%^c^	0	0	50	50	0	100
**2013**						
Samples^a^	11	18	31	8	70	138
Cases^b^	0	0	0	0	1	1
%^c^	0	0	0	0	100	100
TOTAL						
Samples^a^	180	245	592	232	821	2070
CASES^b^	35	46	52	36	290	459
%^c^	7.63	10.02	11.33	7.84	63.18	100

^a^Samples sent for diagnosis to the Unit of Zoonotic Disease.

^b^Laboratory-confirmed canine rabies cases.

^c^Percentage represents proportion of national positive canine rabies cases.

### Social factors

**Fig 3:**
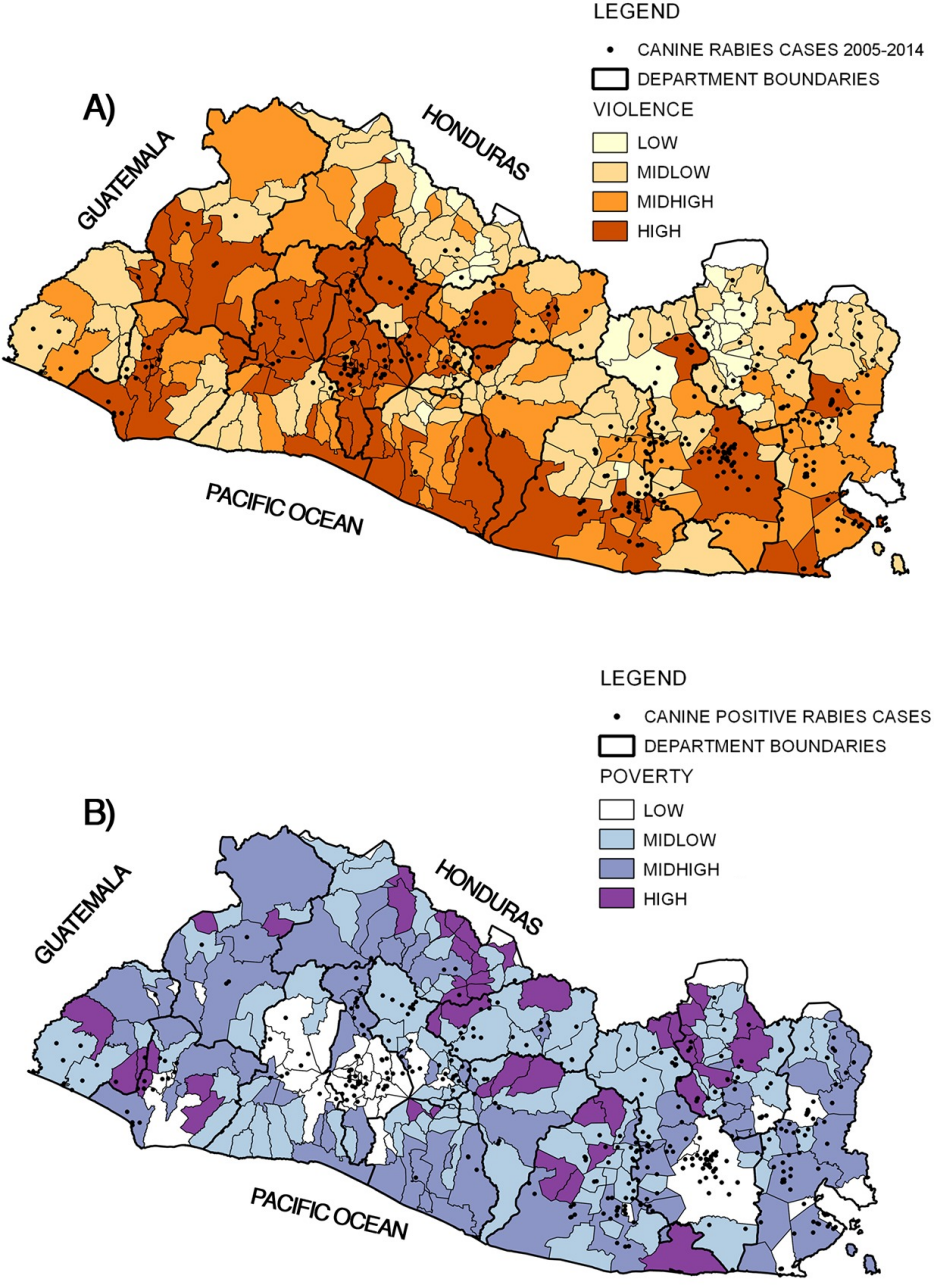
Canine rabies cases in 2005–2014 (points) and social factors indicated in maps. A) Violence classification (low, mid-low, mid-high, high) [25, 56]; B) Poverty classification (low, mid-low, mid-high, high) [24]. Source: Ministry of Health, El Salvador. Use of data authorized by Geoportal-CNR under a CC BY license, with permission from Geoportal-CNR, original copyright 2014.

Violence and poverty classifications were mapped with canine rabies cases, as shown in Fig 3. A majority of the country had a mid-low level of violence (109 townships, Fig 3A), while the highest incidence of canine rabies was observed in townships with mid-high and high levels of violence. The departments with high levels of violence and numbers of positive canine rabies cases were San Miguel (Eastern region), which had 99 positive cases and 55 murders per 100,000 people, and San Salvador (Metropolitan area), which had 52 positive cases and 76 murders per 100,000 people. Both are among the top-five most violent departments. Most townships were categorized as having high poverty (108 townships), but a higher occurrence of canine rabies was observed in low-poverty townships. San Salvador in the San Salvador department (Metropolitan region) and San Miguel in the San Miguel department (Eastern region) are two of the most important townships in the country, and both were classified as having low poverty.

### Factors associated with the incidence of canine rabies

The multivariate analysis (Table 2) revealed that poverty level had a strong, positive association with canine rabies and was the highest risk factor for the disease (low poverty RR = 7.79, p = 0.046). Canine population density (RR = 1.014, p<0.001), the number of public health centers (RR = 1.034, p = 0.002), and illiteracy (RR = 1.045, p = 0.003) were also significantly associated with canine rabies. Finally, violence (mid-high violence RR = 0.66, p = 0.046) had a statistically significant but negative association with canine rabies. However, when univariate linear regression analysis was performed between murder rate and canine rabies cases, a positive correlation was observed (S1 Graph).

**Table 2:** Factors associated with canine rabies, relative risks, and 95% confidence intervals estimated with a Poisson-regression model adjusted for overdispersion in 262 townships, El Salvador, 2005–2014.

Canine Rabies (n = 2620)	RR	β	Std. Err.	P>z	[95% CI]
**Canine population density**	1.014	0.014	0.002	<0.001	1.011–1.018
**Human population density**	0,998	-0.002	0.000	<0.001	0.998–0.999
**Vaccination**	1.004	0.004	0.003	0.265	0.997–1.011
**Health public center**	1.034	0.033	0.011	0.002	1.013–1.055
**Illiteracy**	1.045	0.044	0.015	0.003	1.015–1.075
**Violence**					
Mid-high (656)	0.653	-0.426	0.140	0.046	0.430–0.993
High (654)	0.665	-0.409	0.147	0.064	0.431–1.024
**Poverty**					
High (1080)	2.057	0.721	0.743	0.046	1.014–4.175
Low (350)	7.792	2.053	3.283	0.000	3.412–17,793
Mid-low (730)	2.512	0.921	0.945	0.014	1.201–5.252

Vaccination coverage was a risk factor for canine rabies, but the result was not statistically significant (RR = 1.004, p = 0.265). Human density had a negative association, but this value was very close to the risk threshold (RR = 0.998). The Poisson model regression had a good fit to the data with a deviance df of 0.81 (Table 2).

### Cluster analysis

#### Unadjusted analysis

**Fig 4:**
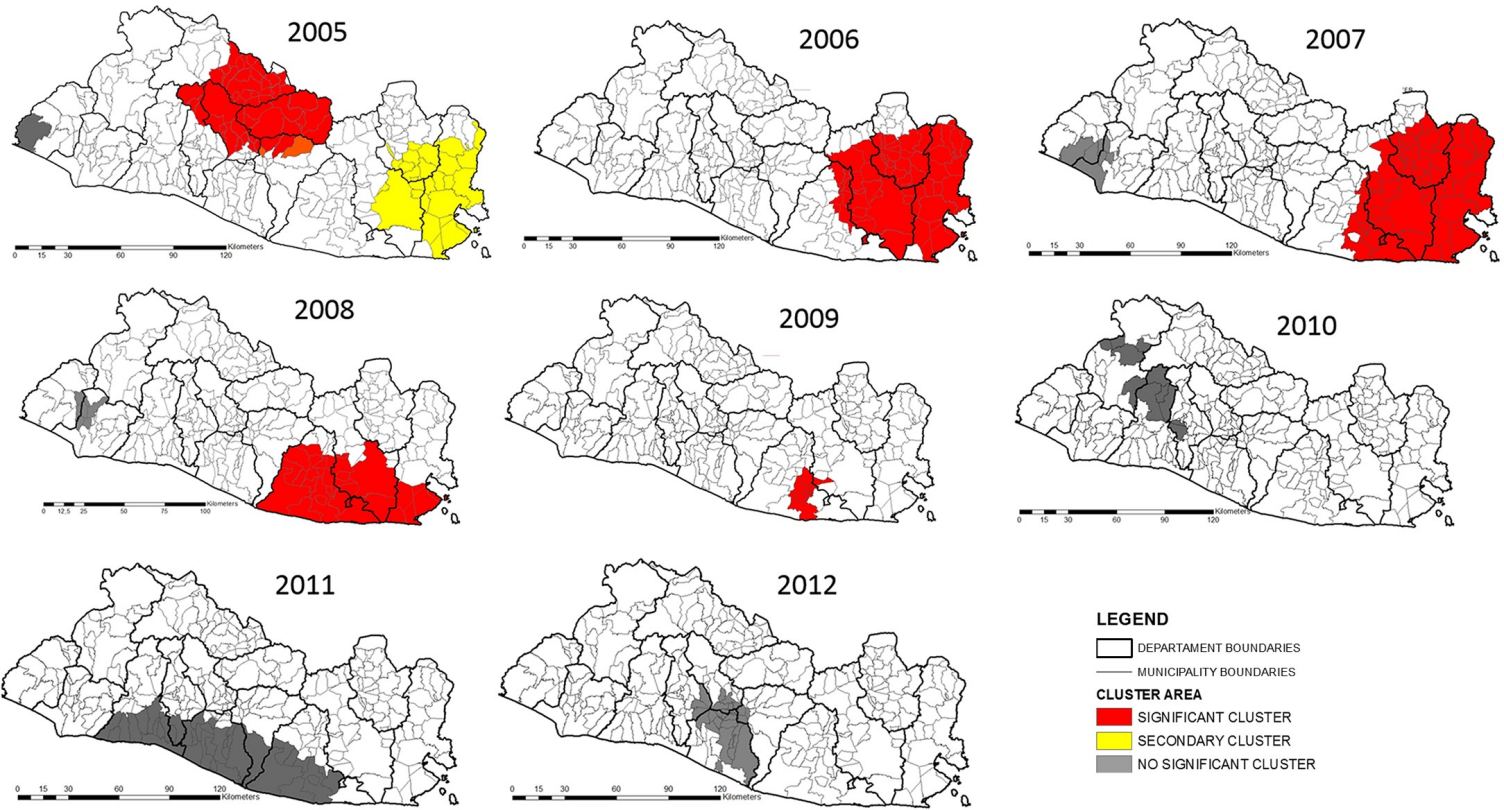
Canine rabies clusters unadjusted for covariates in El Salvador, 2005–2014. Statistically significant clusters (p < 0.05) were identified in the Eastern, Central, Paracentral, and Metropolitan areas; no clusters were detected in 2013, and no positive rabies cases were reported in 2014. Use of data authorized by Geoportal-CNR under a CC BY license, with permission from Geoportal-CNR, original copyright 2014.

For the cluster analysis unadjusted for covariates, 10 significant clusters of canine rabies were detected within the 10-year study period, which were identified from 2005 to 2009 (Table 3). Some of the clusters overlapped. The highest relative risk cluster was located in the Eastern region in the department of Usulutan (RR = 50.62, p < 0.05). The Eastern region had significant clustering in 2005–2009. Other clusters were in the Central, Metropolitan, and Paracentral townships. No significant clusters were found after 2010 (Fig 4).

**Table 3:** Significant cluster location (p *<* 0.05) of canine rabies, El Salvador, 2005–2014. Clusters are shown for adjusted and unadjusted models for covariates.

		Unadjusted				Adjusted ^a^		
Year	Cluster location	Number of cases^b^	*p-value*	RR	Cluster location	Number of cases^b^	*p-value*	RR
**2005**	Central Region and Metropolitan area	53	<0.001	6.49	Central Region, Metropolitan area and Paracentral	40	<0.001	8.09
** **	Central Region and Paracentral	32	<0.001	7.51				
** **	Eastern Region	42	<0.001	4.76				
** **	Eastern Region	15	<0.001	15.23				
** **	Central Region	21	0.003	4.08				
** **	Eastern Region	21	0.039	3.81				
**2006**	Eastern Region	140	<0.001	16.98	Eastern Region	81	<0.001	6.20
** **					Eastern Region	64	<0.001	6.68
**2007**	Eastern Region	47	<0.001	5.09	Eastern Region	19	<0.001	7.98
** **					Eastern Region	11	<0.001	10.38
** **					Eastern Region	11	0.031	7.52
** **					Western Region	6	0.043	11.28
**2008**	Eastern Region	19	0.005	4.84	Western Region	6	0.006	17.26
** **					Eastern Region	4	0.021	5.33
**2009**	Eastern Region	10	<0.001	50.62	Eastern Region	12	<0.001	50.93

^a^ Covariates: violence, poverty, illiteracy, canine population density, human population density, number of public health centers and vaccination coverage

^b^ Canine rabies positive cases laboratory-confirmed

#### Adjusted analysis with covariates

**Fig 5:**
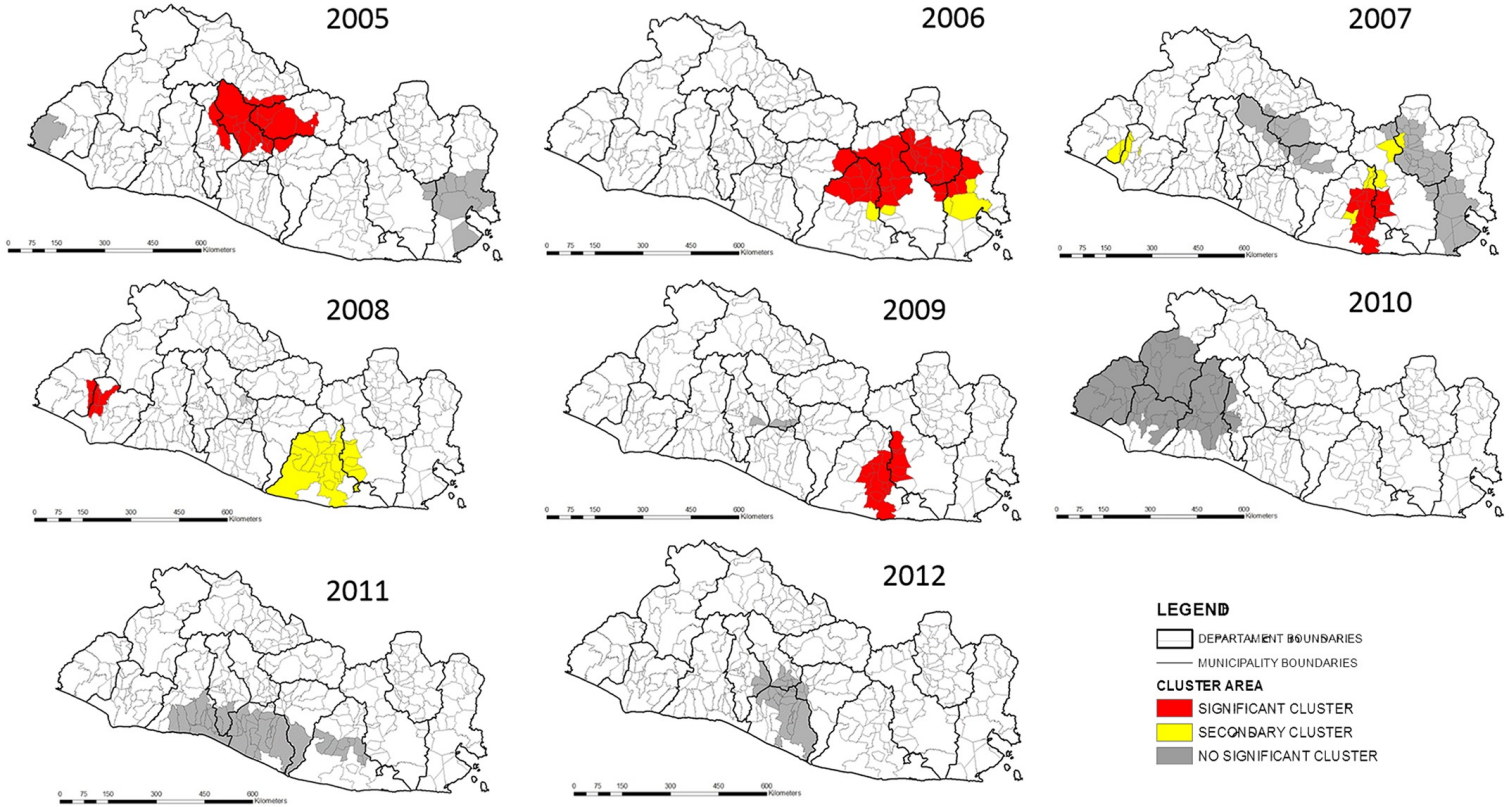
Canine rabies clusters adjusted for covariates in El Salvador 2005–2014. Adjustments were made using several variables (violence, poverty, illiteracy, canine population density, human population density, number of public health centers, and vaccination coverage). Statistically significant clusters (p < 0.05) were found in all five epidemiological regions; no clusters were detected in 2013, and no positive rabies cases were reported in 2014. Use of data authorized by Geoportal-CNR under a CC BY license, with permission from Geoportal-CNR, original copyright 2014.

For the cluster analysis adjusted for covariates (violence, poverty, illiteracy, canine population density, human population density, number of public health centers, and vaccination coverage), 10 significant canine rabies clusters were detected from 2005 to 2009. Overlapping clusters were found with different relative risks. The highest relative risk was identified in the Eastern region, as in the unadjusted analysis (RR = 50.93, p < 0.05) (Fig 5).

In 2005, the Eastern cluster completely disappeared, although persistent rabies clusters were detected for the years 2006–2009. Those later clusters were smaller in size. The Central region cluster remained but was smaller than in the unadjusted analysis. A new cluster was identified in the Western region in 2007–2008. Finally, no change was observed in the Usulutan department cluster in 2009, as in the unadjusted analysis.

### Risk assessment

A risk map was created with the expected number of canine rabies cases/km^2^, which was estimated with a Poisson regression and the independent variables. described above: violence, poverty, illiteracy, canine population density, human population density, number of public health centers and vaccination coverage

**Fig 6:**
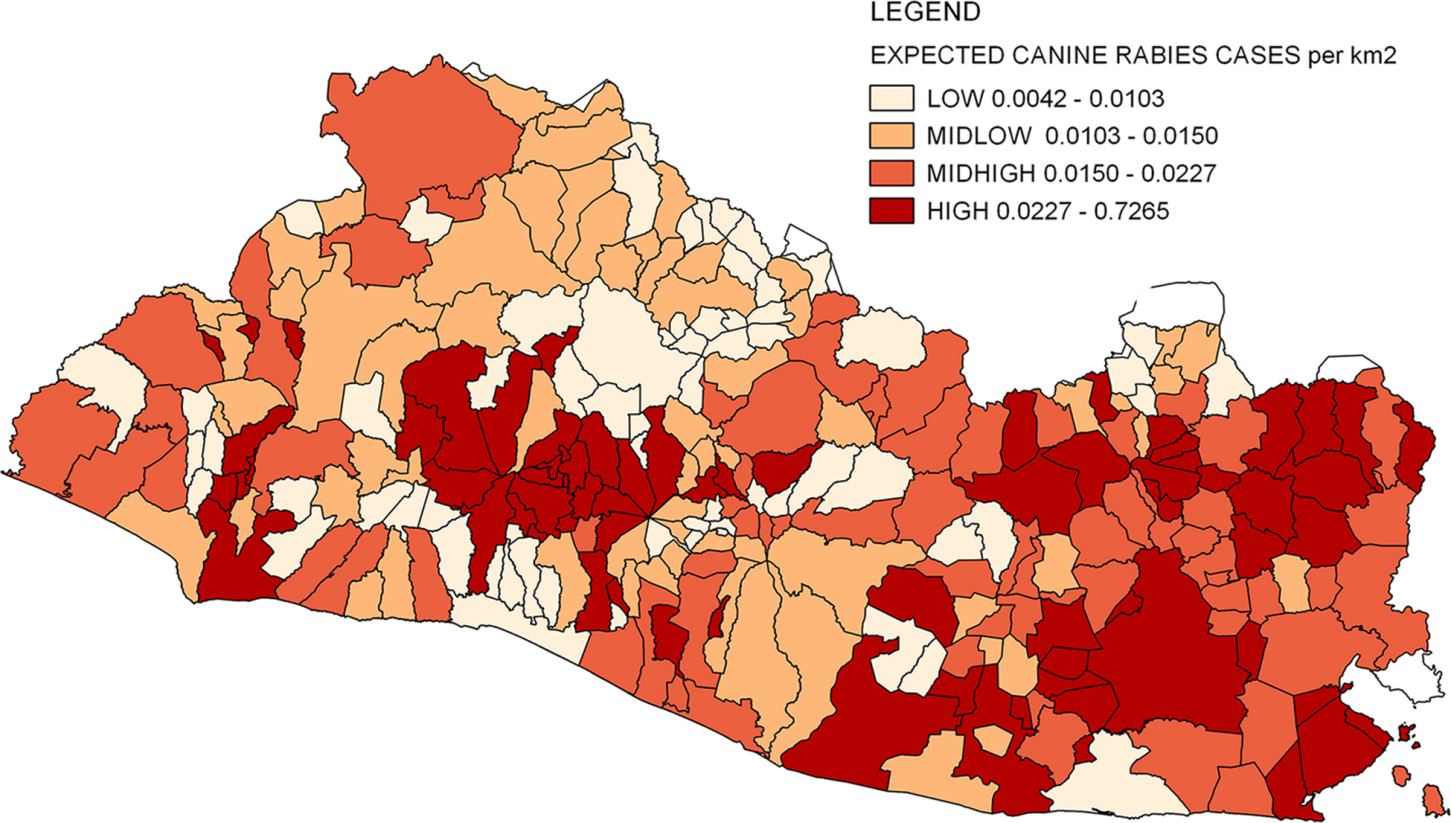
Canine rabies risk assessment. Expected canine rabies cases per km^2^ were estimated with Poisson-regression analysis including the variables violence, poverty, illiteracy, canine population density, human population density, number of public health centers, and vaccination coverage. Use of data authorized by Geoportal-CNR under a CC BY license, with permission from Geoportal-CNR, original copyright 2014.

Out of the 262 townships, 66 were classified as having high risk, which comprised 47% (31) in the Eastern region, 21% (14) in the Metropolitan area, 14% (9) in the Central region, 11% (7) in the Eastern region, and 8% (5) in the Paracentral region. Of those 66 townships, 40% (27) were classified as having low poverty and large urban areas. These were mostly in the Metropolitan, Central, and Eastern region, such as San Miguel. The Eastern region had the highest number of positive cases of canine rabies during the study period study (Fig 6). The same hot spots were identified in the Eastern, Metropolitan, Central, and Western regions in the Kernel density analysis.

## Discussion

This study analyzed social, demographic, and surveillance variables as determinants of canine rabies and their influence on its spatial distribution in El Salvador between 2005 and 2014. Two persistent canine rabies clusters were detected in the Eastern and Central region throughout the study period. The adjusted cluster analysis showed significant differences in cluster size in comparison with the unadjusted analysis, which confirms the influence of the selected factors in the canine rabies spatial distribution (violence, poverty, illiteracy, canine population density, human population density, number of public health centers, and vaccination coverage). High-risk canine rabies areas were identified in the Eastern, Metropolitan, Central, and Western regions, which were correlated with the hot spots found in the spatial analysis.

Poverty has previously been associated with the occurrence of canine rabies in developing countries [34, 35]. Furthermore, a higher occurrence of rabies cases has been described before in low-income areas on the outskirts of large urban cities in Latin America [6]. In our poverty classification, low poverty means a family lives under the poverty line and live in a place where basic resources are scarce, but they still have easier access to some basic services. On the other hand, mid-high and high poverty conditions are typical of rural areas where the access to basic services is very limited, and income is lower.

The statistical analysis revealed that low-poverty areas (RR = 7.79) had the highest risk of canine rabies (S3 Fig). This finding could be due to characteristics associated to low-income townships which prevailed in urban cities, called urban slums (i.e., San Salvador and San Miguel). These areas are characterized by social exclusion, high density of street canines, overcrowding and urban gang violence [36]. All these characteristics could be an impediment to control activities such as, vaccination campaigns or surveillance, hence facilitating the spread of a canine rabies outbreaks [5]. Most importantly, it reflects how health inequalities can define the profile of a disease or the difference in surveillance activities within a specific area.

The results also suggest that the number of public health centers is a significant factor for rabies prevention and control (RR = 1.034). The lack of health facilities in rural areas, where moderate and severe poverty prevails, can lead to a decrease in surveillance sensitivity, unreported cases, and unavailability of post-exposure prophylaxis for humans [21], which lead to an epidemiological silence (Fig 2). Levels of illiteracy are a significant risk factor associated with canine rabies (RR = 1.045). Vulnerable populations not only live in poverty but also have low rabies awareness. The lack of rabies awareness in the population could be a reason for low participation in vaccination campaigns and result in unreported cases of canine rabies [37, 38].

Violence has not been previously associated with rabies. However, in this study, we selected it as a social determinant because of its influence in the social context of El Salvador. Gang violence is a problem that affects the whole country in both urban and rural areas [25]. For example, in a rural area, if a person has been bitten by a dog but his house and health center are in different gang territories, he would need to ask for permission from the gang members to cross to the other side, or his life would be in danger. This could lead to under-reporting of suspected cases. Furthermore, access to health workers is also limited to an insufficient time (perhaps a couple of hours), or even denied. These factors could seriously compromise the implementation of control programs or surveillance activities [14].

In the statistical analysis, mid-high violence had a significant p value but a negative association with rabies (RR = 0.665), although the correlation analysis had a positive slope (S1 Graph). Such results may be influenced by reporting bias caused by limited access to gang areas, as shown in S2 Graph. A higher murder rate correlated with a lower number of suspected cases of rabies, indicating a possible role of violence as a barrier for epidemiological activities. Areas without rabies prevention activities or surveillance could become risk zones for rabies with epidemiological silence. Canine population density was found to be another important significant risk factor for (RR = 1.014). In areas with high canine population density, the probability of dog-to-dog contact is increased, facilitating viral spread among the canine population (S1 and S7 Figs) [37].

In the cluster analysis, a persistent statistically significant hotspot was identified within the Eastern region in 2006–2009. This region had the highest number of canine rabies cases during the study period and is historically recognized as a high endemic area for the disease [7]. Since 2006, health authorities reported vaccination coverage above 80%, and in 2010, vaccination campaigns started along the Honduras border [39]. The improvement of vaccination coverage over time coincides with reductions in the cluster size during the study period, and the cluster became insignificant in 2010 (Fig 5). Furthermore, the Central region cluster of 2005 was identified in the adjusted and unadjusted analyses.

For the adjusted cluster analysis, it is important to highlight the disappearance of the canine rabies cluster identified in the Eastern region for the year 2005 (Fig 5.), which was present in the unadjusted cluster analysis (Fig 4). This result and the downsizing of persistent outbreaks of canine rabies observed in the Central and Eastern regions confirm the influence of violence, poverty, illiteracy, canine population density, human population density, number of public health centers, and vaccination coverage on the spread and persistence of canine rabies. With the analytical approach used in this study, a new rabies focus was identified in a cluster found in the Western region in 2008–2009. These clusters and the persistence of several rabies foci need further analysis to identify the currently unknown rabies factors for these areas and to explore customized strategies for its control.

Lastly, in the risk map, higher risk of canine rabies areas was found in the Metropolitan, Central, Eastern, and Western regions, which were also identified with the kernel density analysis and the spatial scan statistics. For the spatial analyses, the influence of spatial dependence and the edge effect had to be considered [23]. Adjustments for overdispersion were included to compensate for spatial dependence, and the optimized maximum likelihood was included in the statistical analysis [40]. Edge effects cannot be excluded due to the administrative boundaries in El Salvador, especially with canine rabies present in neighboring countries [23]. Migration data from those countries could be included to assess the potential spatial effects. Based on the study results, there is a need to highlight the importance of implementing education programs in vulnerable populations to increase rabies awareness and facilitate control interventions [17, 37]. In addition, it is crucial to standardize the collection of surveillance data in all epidemiological regions to promote communication and institutional coordination.

However, some limitations of the study need to be noted. For example, the association between canine vaccination coverage and positive cases of canine rabies was not statistically significant. This result could be due to a limitation in the method used for the estimation of canine population which, in turn, affects the calculation on the needed vaccination coverage [41]. Furthermore, the data available for illiteracy and human demographics taken from the 2007 population census may not be accurate in population numbers for some difficult access areas, which could be amplified by the population estimation methodology used by the national census authority [16].

The data collected in this study were selected while considering three characteristics: they were collected at the same resolution level, they have epidemiological importance in rabies control, and they adequately represent poverty and violence for the purpose of the study. Because of the limitations in the data collection, we selected data from past years or different resolution levels, but they are important and representative for the present analysis.

Possible reporting bias in the study could have been influenced by three factors: a) the heterogeneity distribution of the human population, which could lead to underestimation of the risk in lower-populated areas; b) highly violent areas with limited surveillance activities; and c) the number of public health centers in rural areas, which could have affected the sensitivity in the surveillance analysis. Adjusting for these variables is expected to remove the effect of this bias.

Overall, the high-risk areas identified in El Salvador correlated well with the hot spots found with the scan statistics results with further cluster analysis adjusted for the social, demographic, and surveillance variables included in the study. Such results could provide useful information to health authorities and assist in the decision-making process for the development of new intervention strategies for rabies control and prevention. The results could also help to prioritize both identified risk areas and those with the highest levels of violence.

The findings can be applied in public health planning for vulnerable communities where rabies is a critical public health problem. Social features could be key in the development of targeted intervention strategies directed towards those vulnerable populations and other areas with similar social conflict, such as other Latin American countries, Asia [42], and Africa [43].

Finally, this approach of identifying social determinants could be applied to other infectious diseases, primarily those related to poverty, such as human immunodeficiency virus (HIV), tuberculosis (TB), or dengue [44, 45]. For example, HIV/AIDS and TB deaths have increased to 1.5 million and 1.2 million [46], respectively, and they disproportionately affect the poorest populations. The conditions and environment in which poor communities live can facilitate the transmission. Reaching vulnerable communities with weak health systems and limited access should be valuable for applying an integral approach that includes education for disease prevention.

## Conclusion

The findings of this study suggest an association between canine rabies and its spatial patterns with social determinants in El Salvador, such as level of poverty, violence, illiteracy, and demographic factors. This novel approach could be useful for disease prevention and control in developing countries where social crises and rabies coexist. The strategy could also be used to address determinant factors for the persistence of canine rabies.

## Supporting information

S1 Graph Rabies positive cases vs murder rate per 10000 hab. (TIF)

S2 Graph Rabies suspect brain canine samples for laboratory diagnosis vs murder rate per 100,000 hab. (TIF)

S3 Graph Canine brain samples send to diagnosis and laboratory confirmed canine rabies cases per department (2005–2012). (TIF)

S4 Graph Canine rabies cases and mass vaccination coverage by year in El Salvador. (TIF)

S1 Fig Human population density and canine population density in El Salvador. (TIF)

S2 Fig Health public center and canine rabies cases 2005–2014 in El Salvador. (TIF)

S3 Fig Poverty map and Canine rabies cases (black dots); blue circle (San Salvador, Capital city and San Miguel in Western region) higher rabies cases and low poverty areas. (TIF)

S4 Fig Violence map and Canine rabies cases (black dots); (blue circle) townships with highest canine rabies cases and high homicide rate (blue circle). (TIF)

S5 Fig Vaccination coverage in El Salvador during 2005–2014. (TIF)

S6 Fig Canine rabies cold spots in El Salvador 2005–2014 for space-time permutation analysis detecting lowest rabies of canine rabies. (TIF)

S7 Fig Canine rabies estimated risk areas and canine population density in El Salvador. (TIFF)

S1 Excel Canine rabies cases per year and department between 2005–2014 in El Salvador. (XLSX)
