# Supplementary figures and images for: Spatiotemporal analysis of canine rabies in El Salvador: Violence and poverty as social factors of canine rabies
Source: PLoS One. 2018 Aug 17;13(8):e0201305. doi: 10.1371/journal.pone.0201305 (PMC6097665; doi:10.1371/journal.pone.0201305)

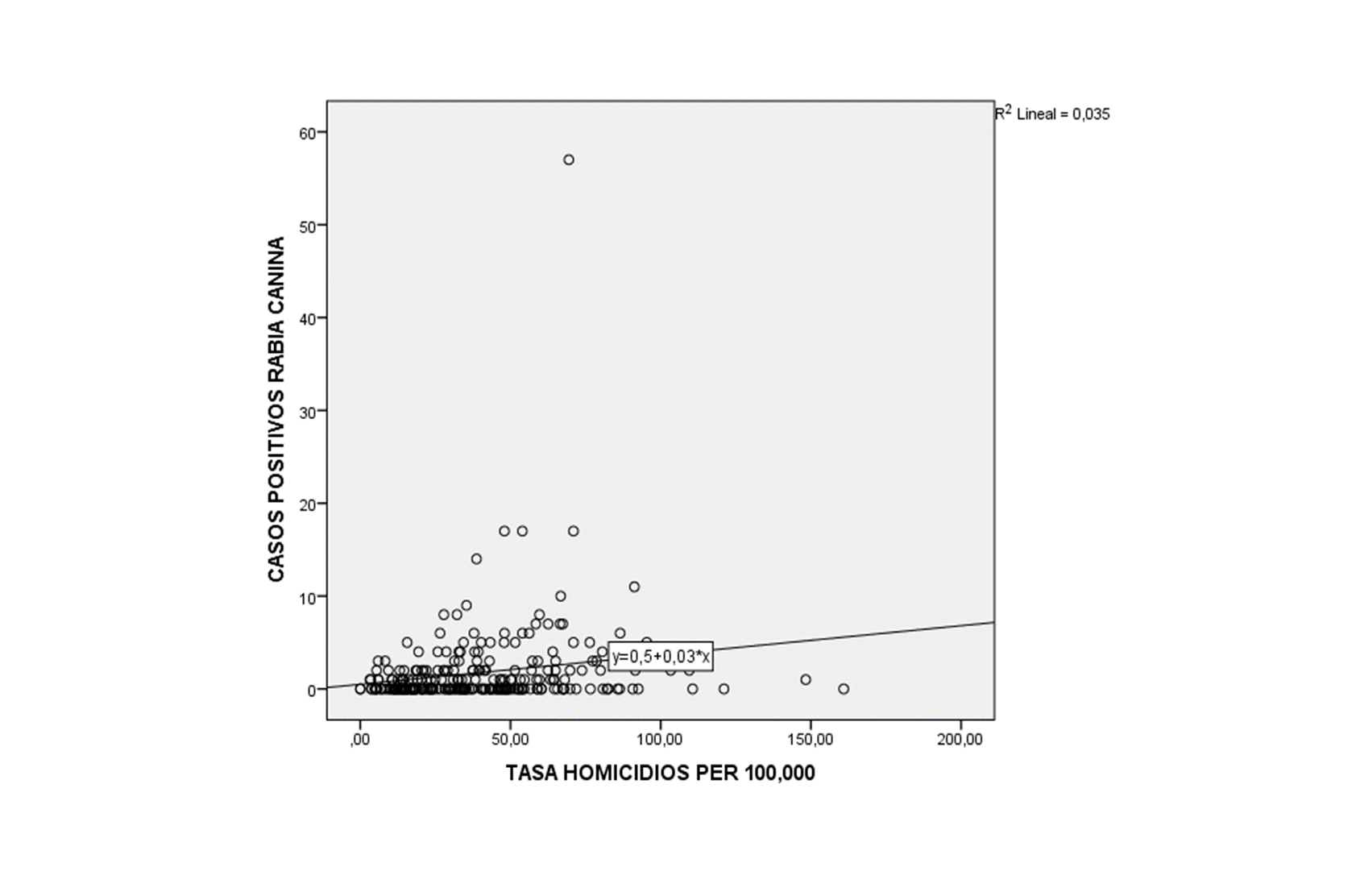

Supplement: S1 Graph — (TIF) [file pone.0201305.s001.tif]

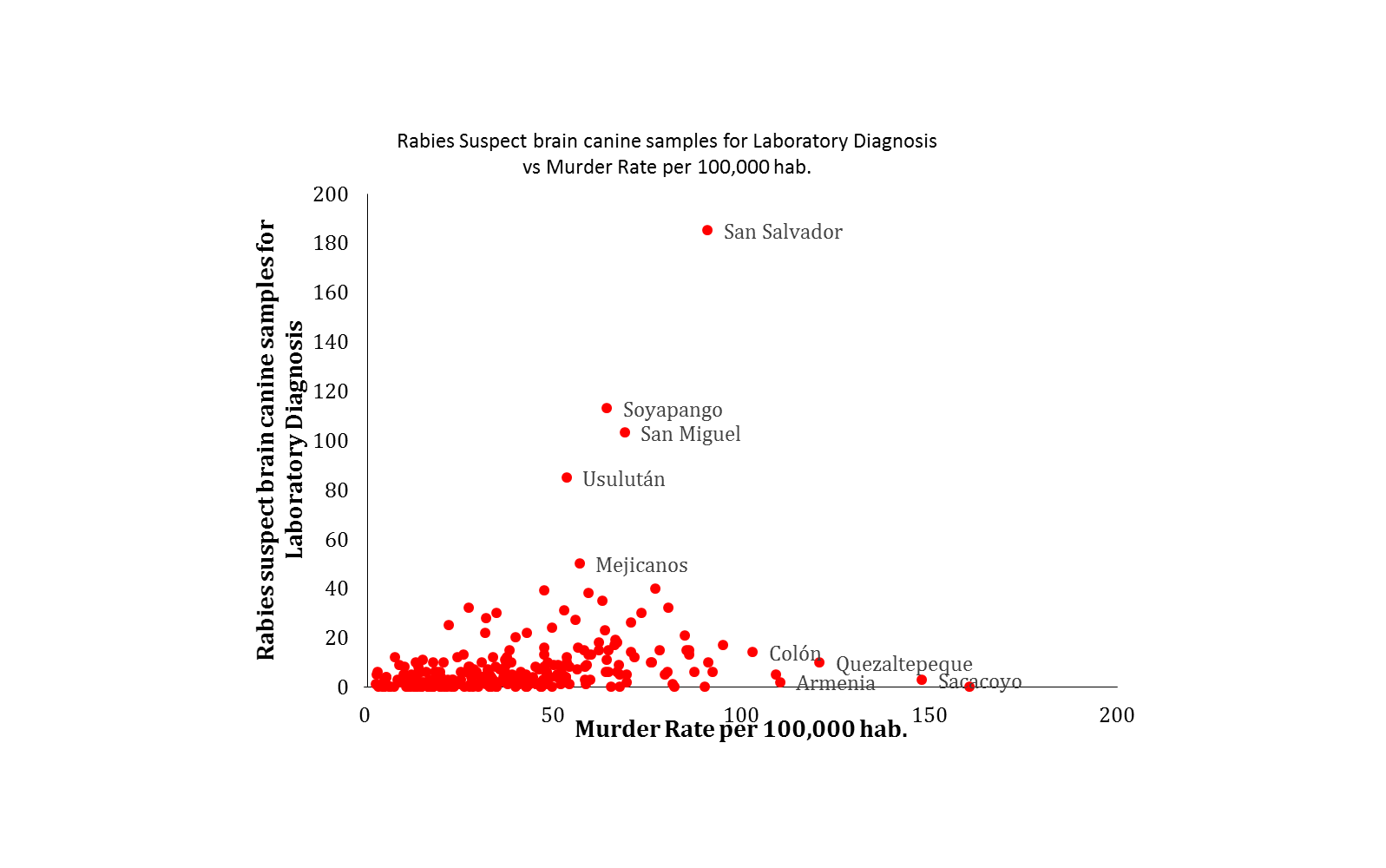

Supplement: S2 Graph — (TIF) [file pone.0201305.s002.tif]

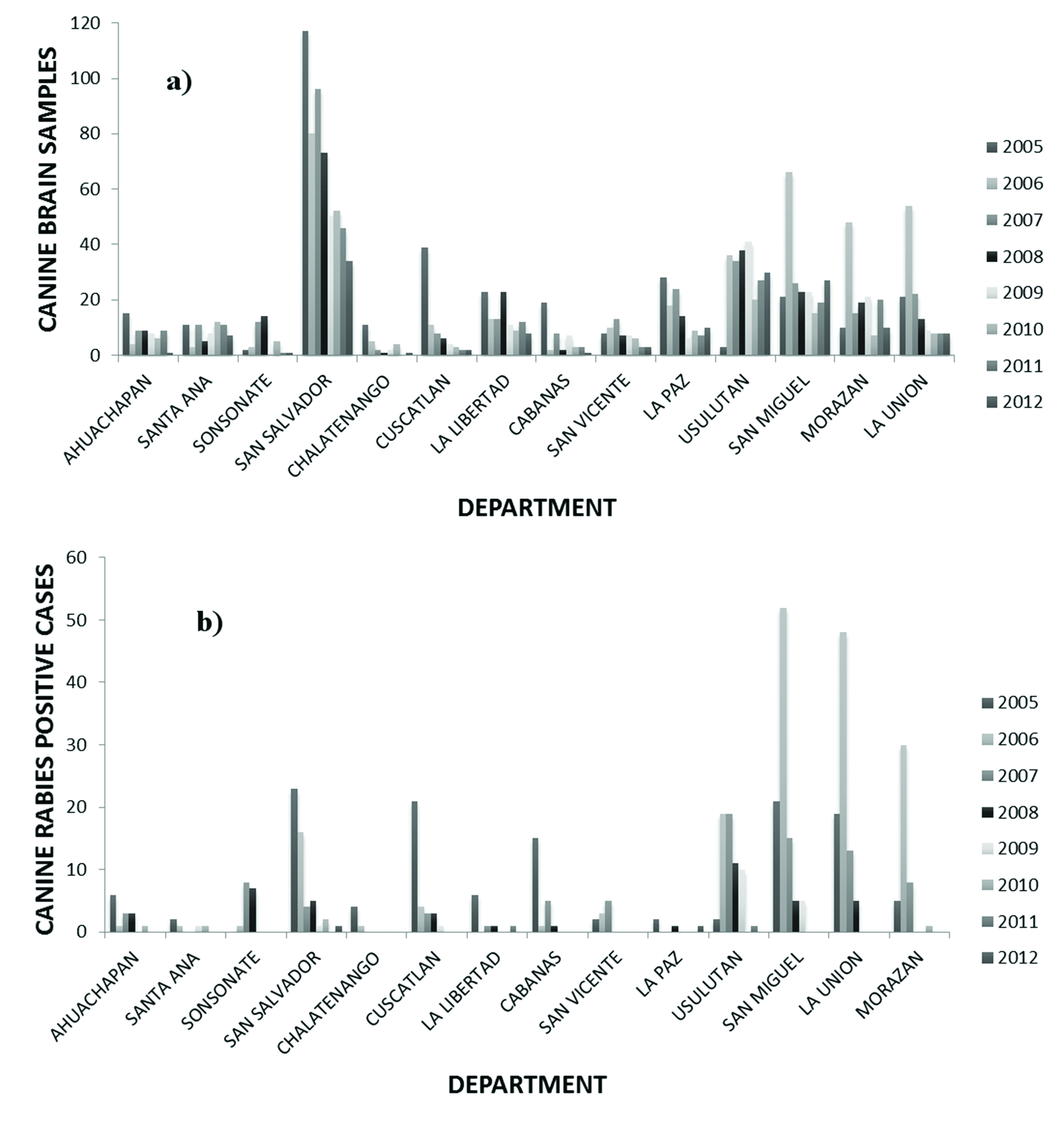

Supplement: S3 Graph — (TIF) [file pone.0201305.s003.tif]

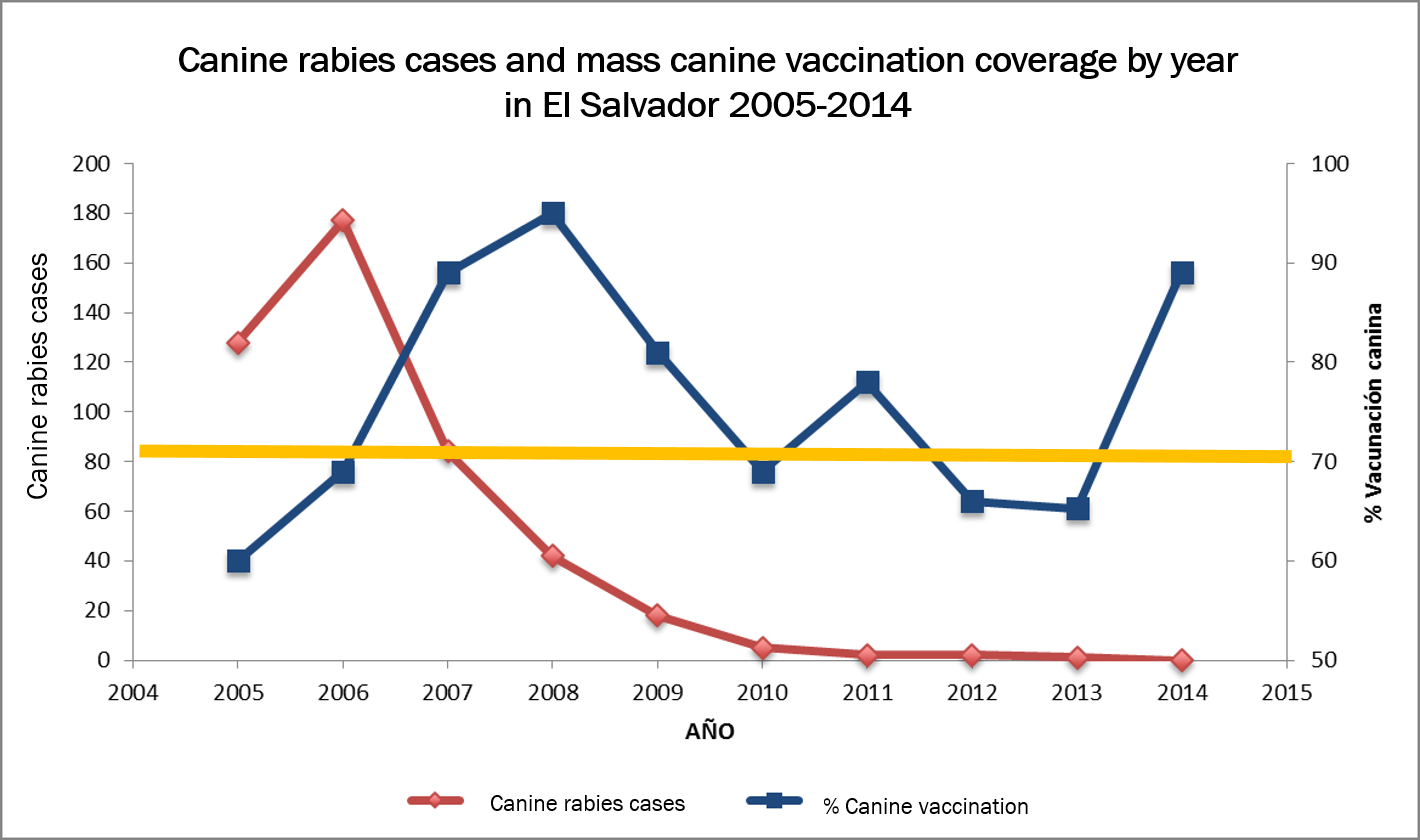

Supplement: S4 Graph — (TIF) [file pone.0201305.s004.tif]

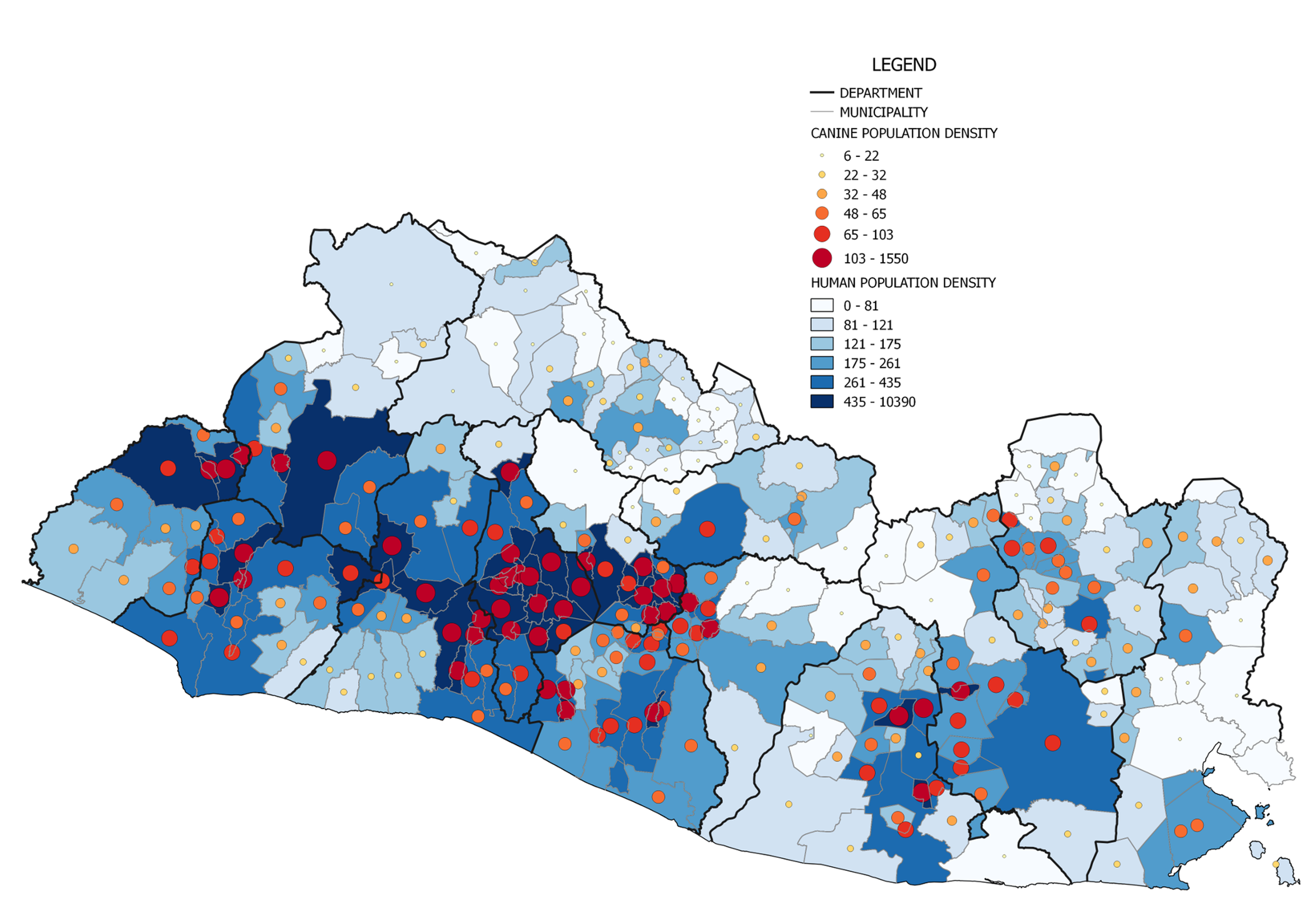

Supplement: S1 Fig — (TIF) [file pone.0201305.s005.tif]

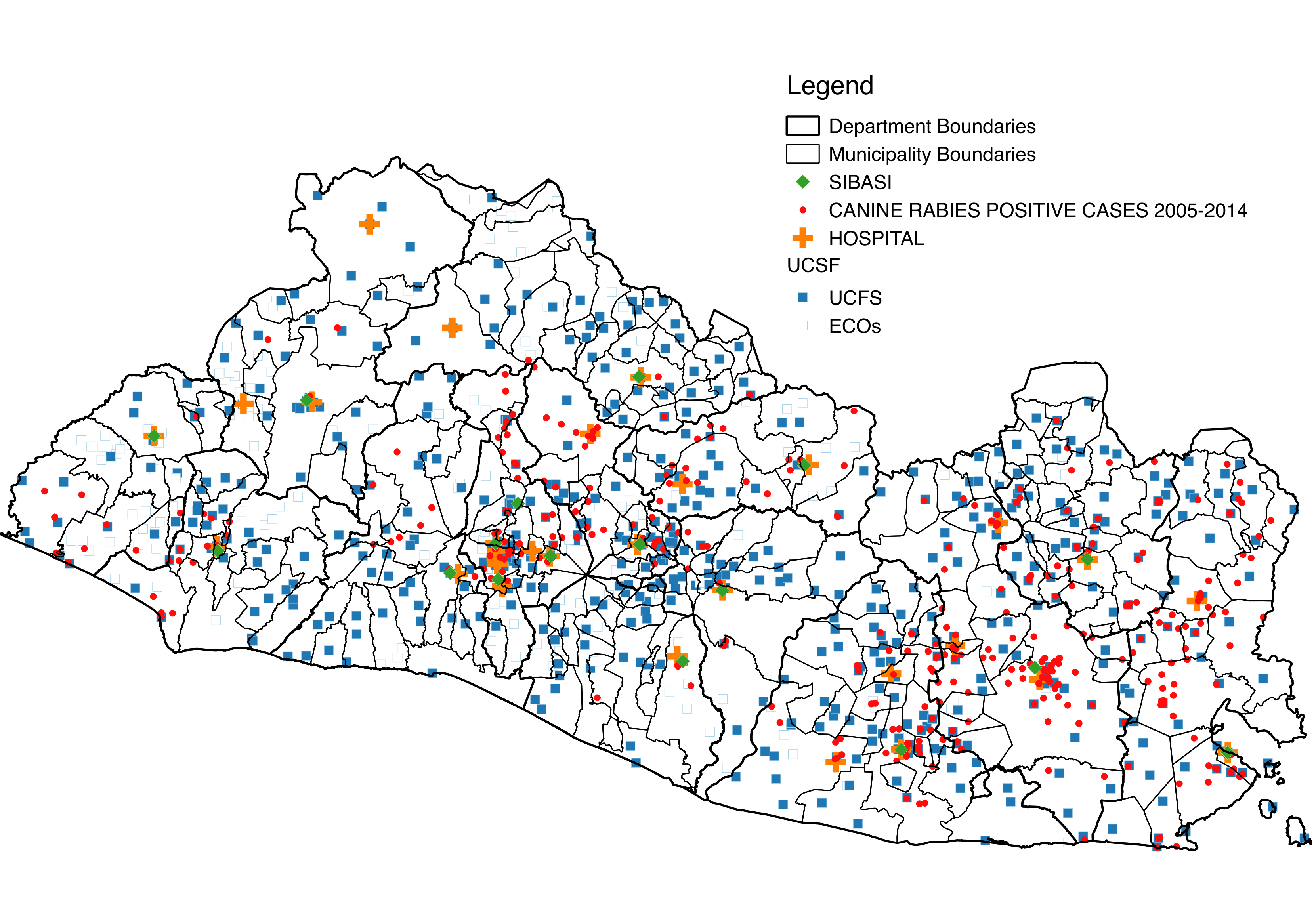

Supplement: S2 Fig — (TIF) [file pone.0201305.s006.tif]

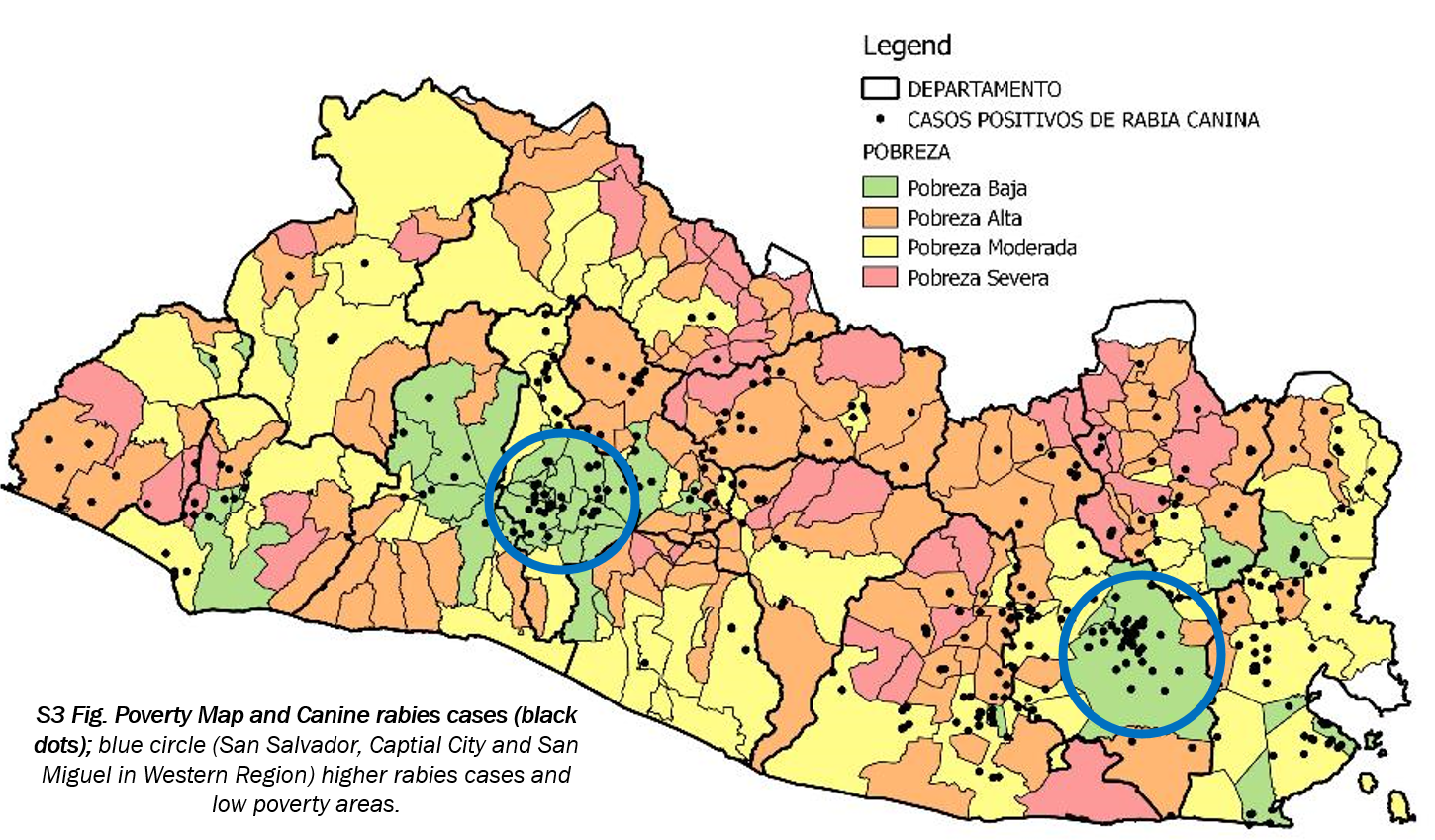

Supplement: S3 Fig — (TIF) [file pone.0201305.s007.tif]

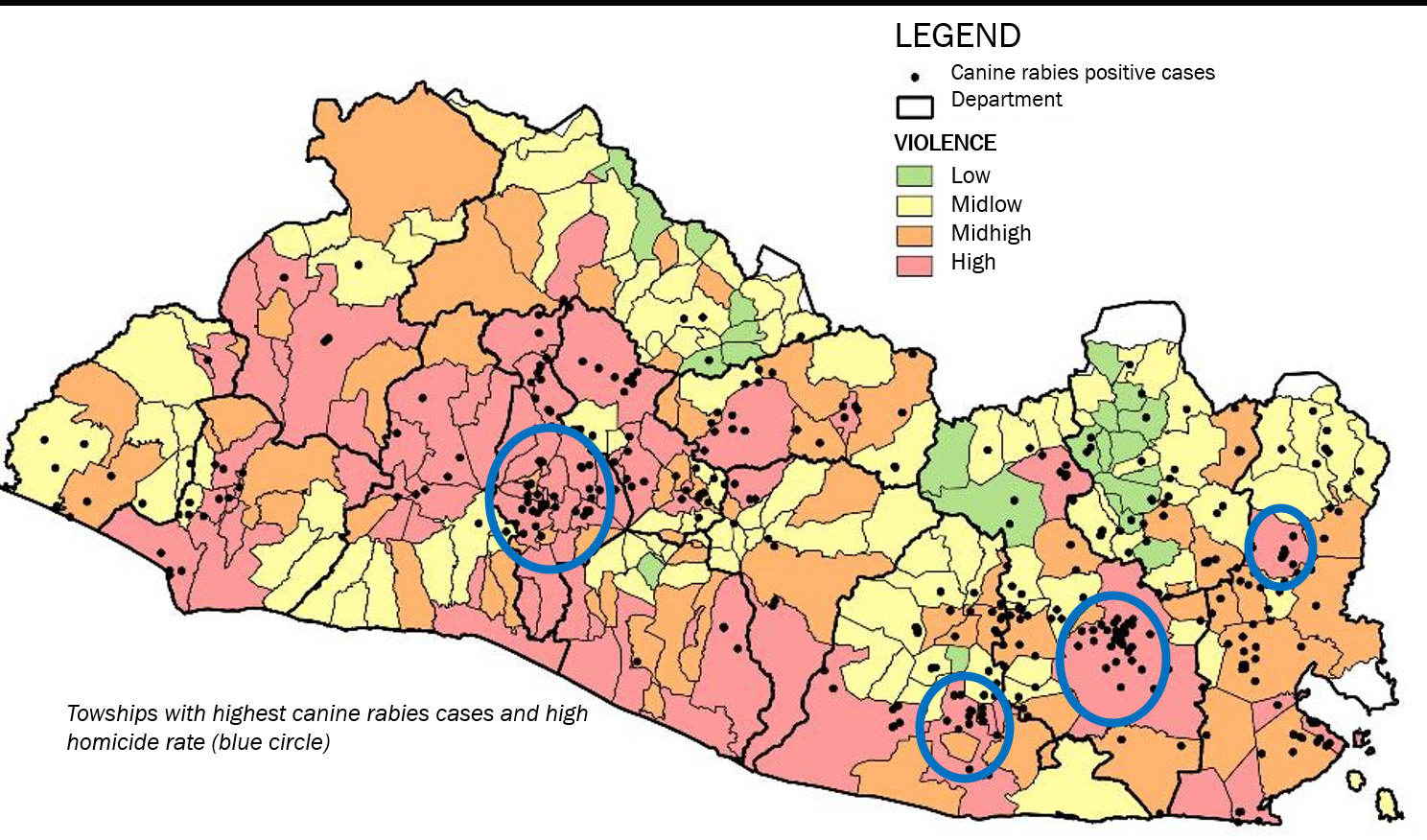

Supplement: S4 Fig — (TIF) [file pone.0201305.s008.tif]

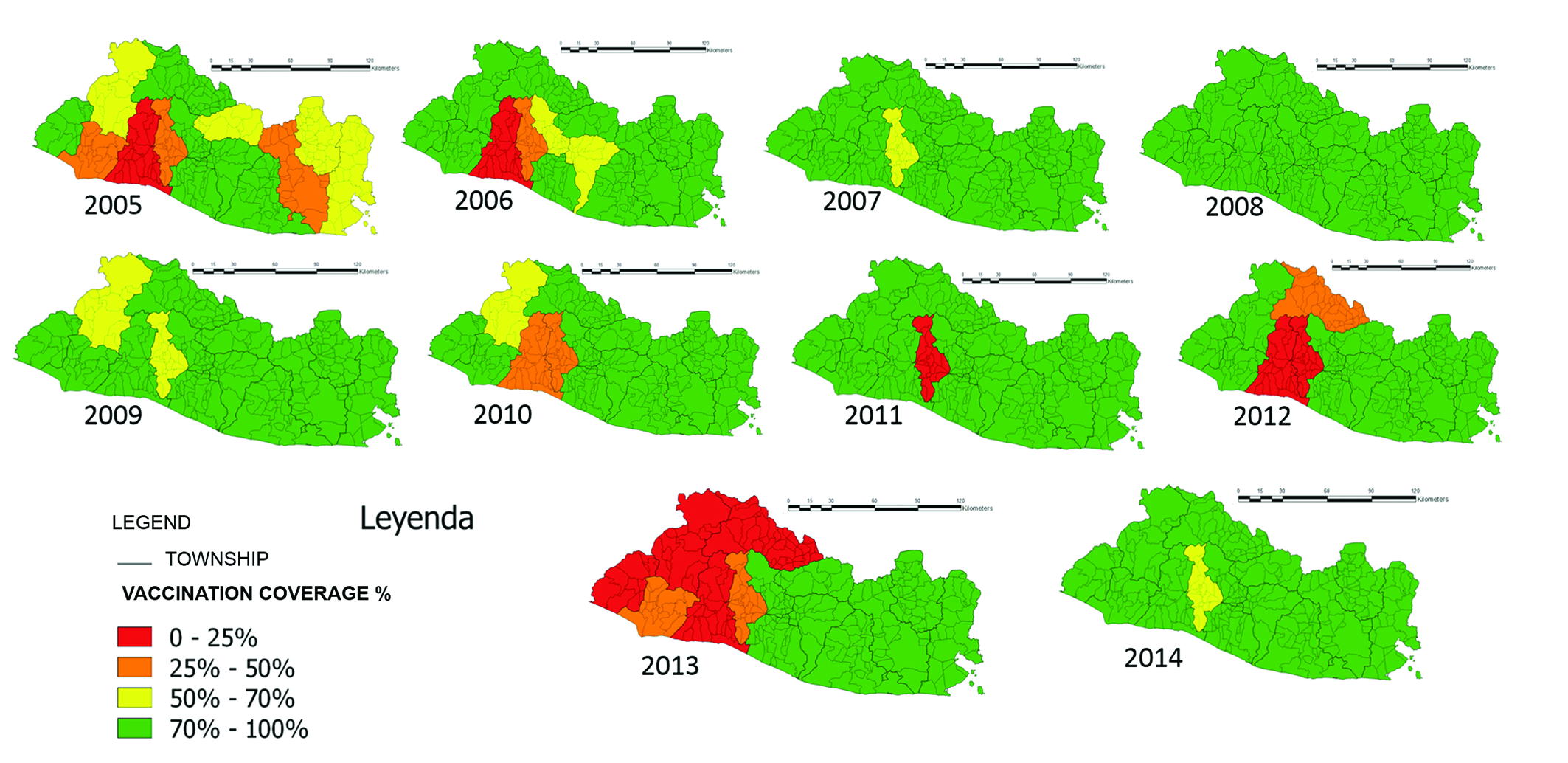

Supplement: S5 Fig — (TIF) [file pone.0201305.s009.tif]

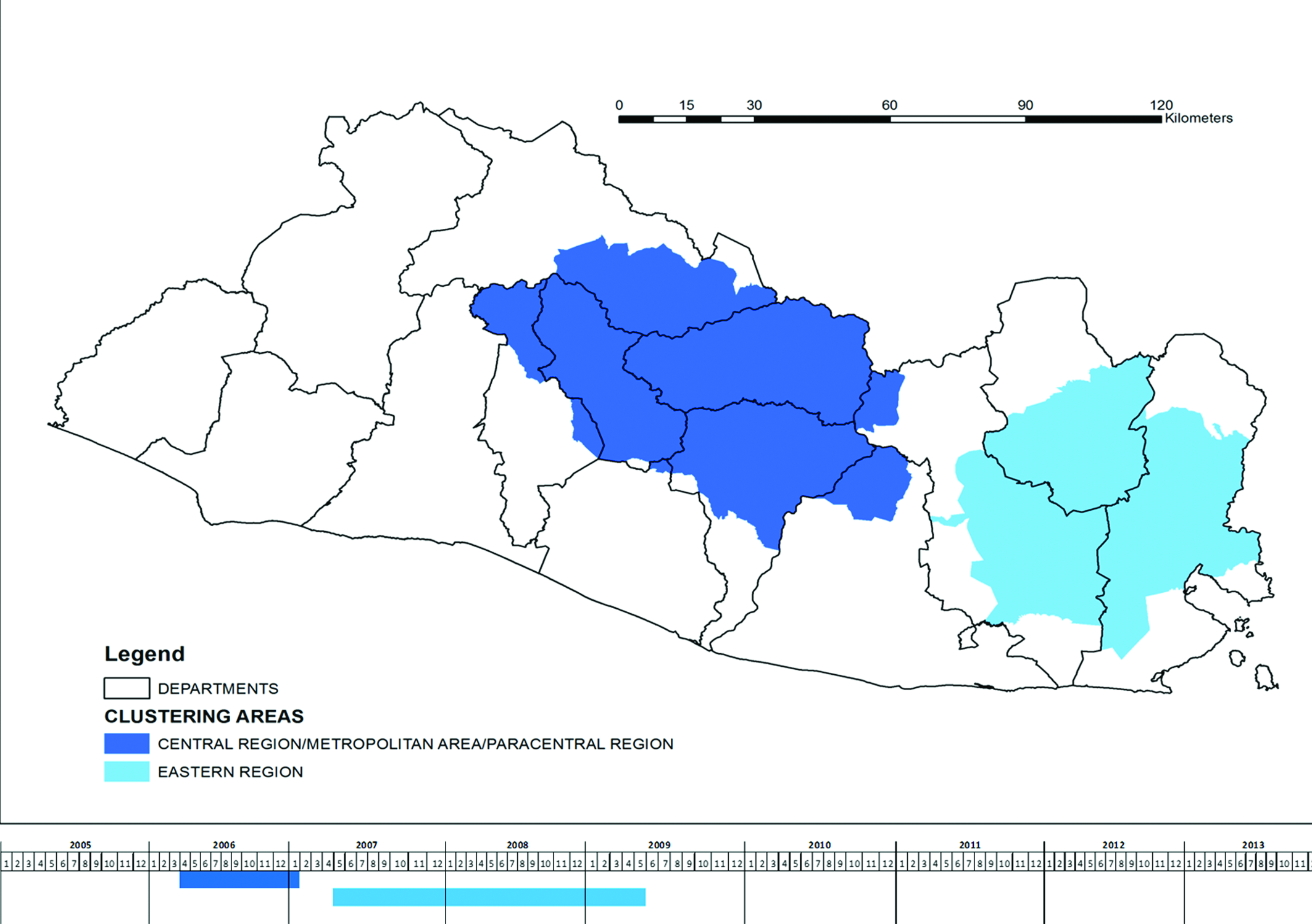

Supplement: S6 Fig — (TIF) [file pone.0201305.s010.tif]
